# Reduction of oxidative stress improves insulin signaling in cardiac tissue of obese mice

**DOI:** 10.31744/einstein_journal/2020AO5022

**Published:** 2020-03-13

**Authors:** Matheus Scarpatto Rodrigues, Bruno Luiz da Silva Pieri, Gustavo de Bem Silveira, Rubya Pereira Zaccaron, Ligia Milanez Venturini, Vitor Hugo Comin, Karine Damian Luiz, Paulo Cesar Lock Silveira

**Affiliations:** 1 Universidade do Extremo Sul Catarinense CriciúmaSC Brazil Universidade do Extremo Sul Catarinense , Criciúma , SC , Brazil

**Keywords:** Obesity, Insulin resistance, Oxidative stress, Myocardium, Mice

## Abstract

**Objective:**

To evaluate the effects of oxidative stress on insulin signaling in cardiac tissue of obese mice.

**Methods:**

Thirty Swiss mice were equally divided (n=10) into three groups: Control Group, Obese Group, and Obese Group Treated with N-acetylcysteine. After obesity and insulin resistance were established, the obese mice were treated with N-acetylcysteine at a dose of 50mg/kg daily for 15 days via oral gavage.

**Results:**

Higher blood glucose levels and nitrite and carbonyl contents, and lower protein levels of glutathione peroxidase and phosphorylated protein kinase B were observed in the obese group when compared with their respective control. On the other hand, treatment with N-acetylcysteine was effective in reducing blood glucose levels and nitrite and carbonyl contents, and significantly increased protein levels of glutathione peroxidase and phosphorylated protein kinase B compared to the Obese Group.

**Conclusion:**

Obesity and/or a high-lipid diet may result in oxidative stress and insulin resistance in the heart tissue of obese mice, and the use of N-acetylcysteine as a methodological and therapeutic strategy suggested there is a relation between them.

## INTRODUCTION

Obesity is a worldwide and multiethnic public health problem, which affects men and women of all age groups and social classes. ^( 1 , 2 )^ Epidemiological data reinforce the prevalence of this condition in the world population. According to a survey by the World Health Organization (WHO) from 2016, over 1.9 billion adults suffered from excessive weight, 650 million of whom were obese. ^( 3 )^ Obesity causes important pathophysiological alterations, such as type 2 *diabetes mellitus* , insulin resistance, ^( 4 )^ and cardiovascular diseases and their associated clinical complications. ^( 5 )^

Several strategies have been used to experimentally study obesity, especially the model of induction through a high fat diet (hyperlipid diet). ^( 6 , 7 )^ Mice that underwent such diet presented significant cardiac problems, such as myocardial fibrosis, cardiomyocyte hypertrophy, and a decreased contractile capacity of the heart. ^( 8 )^ Obese mice presented deficits in glucose uptake and a reduction of insulin sensitivity in the myocardium. ^( 9 )^

There may be several mechanisms involved in the occurrence of insulin resistance in the heart, and an elevated oxidative stress may be one of them. Animals treated with the hyperlipidic diet present an overproduction of reactive oxygen species (ROS) in the liver and adipose tissue. ^( 10 )^ The myocardium of mice that are obese due to a high fat diet present an increase in oxidative stress. ^( 11 )^ However, the studies are not conclusive and warrant further investigation.

Strategically, the use of a classic antioxidant could better demonstrate the relation between oxidative stress and insulin action and be very valuable. Therefore, N-acetylcysteine (NAC), a non-enzymatic antioxidant derived from the amino acid cysteine, with the chemical formula C _5_ H _9_ NO _3_ S and molecular weight of 163.2kDa, ^( 12 )^ is a substance that, due to its antioxidant action, can be experimentally used to better demonstrate this relation.

N-acetylcysteine is a compound often employed in clinical practice as a mucolytic agent to treat paracetamol overdoses and prevent free radical generation by toxic substances. ^( 13 )^ Its antioxidant activity is related mainly to the reduction of the extracellular amino acid cystine into the intracellular amino acid cysteine, and to the donation of thiol groups to reduced glutathione. ^( 14 )^ Moreover, NAC can promote the direct neutralization of ROS as the radical hydroxyl and hypochlorous acid, thus preventing the occurrence of oxidative stress and its possible consequences over insulin resistance. ^( 15 )^

## OBJECTIVE

To analyze the increase of oxidative stress and insulin resistance in the myocardium of mice that are obese from a high fat diet. When such an increase is confirmed, the objective is to analyze if the use of N-acetylcysteine shows a causal relation between such mechanisms.

## METHODS

### Ethnic aspects and animal characterization

This study used thirty 45-day-old male Swiss mice from the Animal Research Lab at UNESC. The mice were initially divided into two groups: 10 animals fed with the standard diet for rodents (Control Group) and the remaining 20 animals fed a high-fat diet, after obesity and insulin resistance were proven, were subdivided into two other experimental groups: Obese Group (n=10) and Obese Group Treated with N-acetylcysteine (n=10). All animals were kept in a 12-hour light/dark cycle, an environment with 70% humidity and temperature between 20°C and 22°C, in polyurethane cages with metallic covers (one animal per box) and fed during 12 weeks with standard feed (carbohydrate: 70%; protein: 20%; fat: 10%; total of 3.8kcal/g) or high-fat feed (carbohydrate: 38.5%; protein: 15%; fat: 46.5%; total of 5.4kcal/g) and water ad libitum.

This study was evaluated and approved by the Animal Ethics Committee (AEC) of the *Universidade do Extremo Sul Catarinense* (UNESC), protocol 042/2016-2. All experiments strictly abided by the ethical principles of experimentation with animals.

### Treating the animals with the antioxidant N-acetylcysteine

After obesity and insulin resistance had been induced, the animals who had previously received the high-fat diet were subdivided into two groups: Group OB, obese mice who were given the high-fat diet (n=10); and Group OB + NAC, obese mice treated with NAC for 15 days (n=10). It is worth mentioning that the animals in the latter group only received the antioxidant therapy after obesity and insulin resistance had been duly confirmed through evaluations conducted after three months of the animals being exposed to the high fat diet. N-acetylcysteine was administered once a day though oral gavage (50mg/kg) for 15 days. The study was conducted in February and March 2017. The full duration of the experimental protocol (experimental period) was 14 weeks and 1 day. After 24 hours of the last administration of NAC, the animals were euthanized, and the heart was extracted so that the biochemical techniques could be performed.

### Fasting blood glucose

The test was done at the end of the experimental period. Food was removed 6 hours prior to the test. Blood glucose was measured using a glucometer and results were expressed as mg/dL.

### Insulin tolerance test

The insulin tolerance test was conducted before the antioxidant administration and after the experimental period. The food was removed 6 hours prior to the test, and the first blood withdrawal corresponded to time zero. After that, insulin (2U/kg of body weight) was injected intraperitoneally, and blood samples were collected from the tail at 5, 10, 15, 20, 25, and 30 minutes. Blood glucose was determined by a glucometer. The speed of the glucose decay constant (K _RI_ ) was calculated through the formula 0.693/t _1/2_ . The t _1/2_ of glucose was calculated from the curve of the analysis of the least squares of the concentration of serum glucose, during the phase of linear decay. This test was done to confirm insulin resistance induction in the obese mice and verify a possible improvement in the treated animals.

### Western blotting

After 24 hours of the last NAC administration, the animals were euthanized, and the cardiac apex was extracted and immediately homogenized in a specific buffer with protease and phosphatase inhibitors. The total protein concentration was determined according to the method by Bradford et al. ^( 16 )^ The proteins were resuspended and preserved in Laemmli buffer with 100mmol/L of dithiothreitol (DTT), ^( 17 )^ and immunoblotting was determined with specific antibodies. For that, aliquots with 250µg of protein per sample were applied over polyacrylamide gel (SDS-PAGE) and were later transferred to nitrocellulose membranes via electrophoresis. After that, the membranes were incubated with specific primary antibodies anti-pAKT, glutathione peroxidase (GPx) and beta-actin acquired from Santa Cruz Biotechnology (Santa Cruz, California, USA) under constant agitation and overnight at 4°C. Later, the membranes were incubated in a solution with peroxidase conjugated secondary antibody for 2 hours, at room temperature. After that, the membranes were incubated for 2 minutes in an enzyme substrate and exposed to the X-Ray film on a radiographic development cassette. The intensity of the bands was determined through the reading of the developed radiographs through optic densitometry, with a scanner (HP G2710) and the software ImageJ.

### Dichlorohydrofluorescein

The oxidation of the 2’7’ dichlorohydrofluorescein diacetate (DCFH-DA) through the cells causes difluorescein fluorescence (DCF), which can easily be read in a spectrophotometer. In this essay, 100µL of water and 75µL of DCFH-DA were added to 25µL of sample homogenate, homogenized in vortex and taken into a water bath at 37°C in the dark, for 30 minutes. Separately, a calibration curve was prepared, which used as a standard the DCFH-DA 0.1µM diluted in phosphate buffer/EDTA at pH 7.4 in different concentrations. The samples and the calibration curve were processed in duplicate and protected from light. After 30 minutes, the results were read in the spectrophotometer (525nm excitation and 488nm emission) and results were expressed in nmol of DCF per mg of protein. ^( 18 )^

### Nitric oxide formation indicator

To measure the nitrite content, the samples were incubated with Griess reagent (1% sulfanilamide and 0.1% of N-1 (naphthyl) ethylenediamine) at room temperature, for 10 minutes and absorbance was measured at 540nm. Nitrite content was calculated with base on a standard curve from 0 to 100nM performed with the metabolite sodium nitrite (NaNO2). Results were calculated in µmol nitrite/mg protein. ^( 19 )^

### Protein carbonylation

The carbonyl content was determined spectrophotometrically at 370nm as previously described by Levine et al. ^( 20 )^ The results were calculated as nmol/mg of protein using the molar extinction coefficient of dinitrophenylhydrazones of 22.000M ^-1^ cm ^-1^ .

### Statistical analysis

Results were expressed as mean±standard deviation and analyzed statistically through the variance analysis (ANOVA) follow by the Bonferroni *post hoc* test. Statistical significance was set at p<0.05. Statistical analyses were done through the software GraphPad Prism ^®^ (version 5.00) for Microsoft Windows ^®^ .

## RESULTS

### Body weight and fasting blood glucose

**Figure 1:**
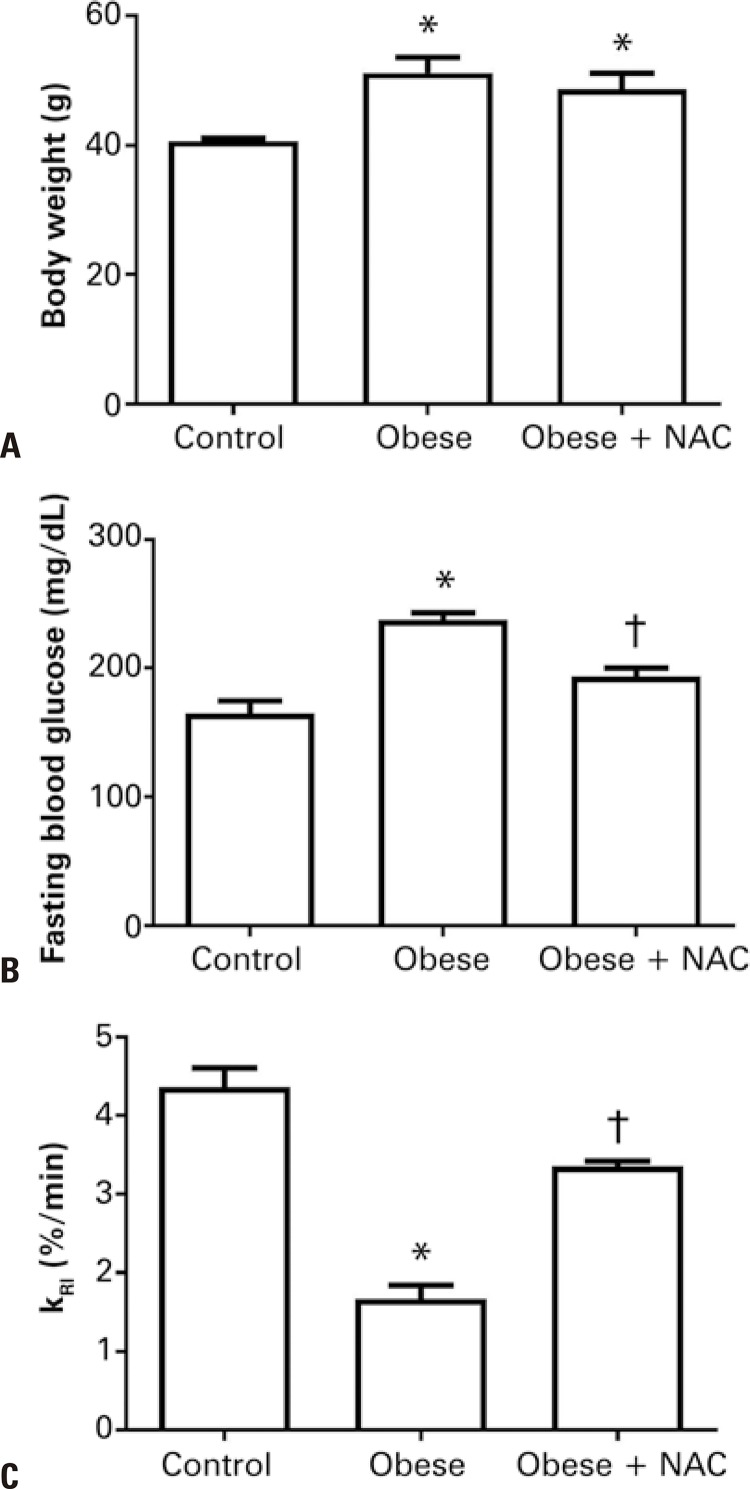
Effects of the supplementation with N-acetylcysteine on body weight, fasting glucose and insulin tolerance in obese mice * p<0.05 *versus* Control Group; ^†^ p<0.05 *versus* Obese Group. NAC: Obese Group Treated with N-acetylcysteine; KRI: speed of the glucose decay constant.

The evaluation of the effects of the NAC supplementation over the body weight, fasting blood glucose and insulin tolerance in obese mice is shown in figure 1 . As expected, the high-fat diet was efficient in increasing the body weight of the mice and administering the antioxidant NAC did not alter this parameter, which remained significantly elevated in relation to the Control Group ( Figure 1A ). The fasting blood glucose levels of the obese mice were higher compared to the Control Group. NAC administration reduced the fasting blood glucose levels in the Group Treated with NAC in relation to the non-treated Obese Group ( Figure 1B ).

### Insulin tolerance test

To evaluate insulin sensitivity among the studied groups, the glucose decay constant (K _RI_ ) was used. Obesity significantly reduced the K _RI_ values in comparison to the Control Group. On the other hand, the treatment with the antioxidant NAC significantly reverted this effect ( Figure 1C ).

### Production of reactive species in the cardiac tissue

**Figure 2:**
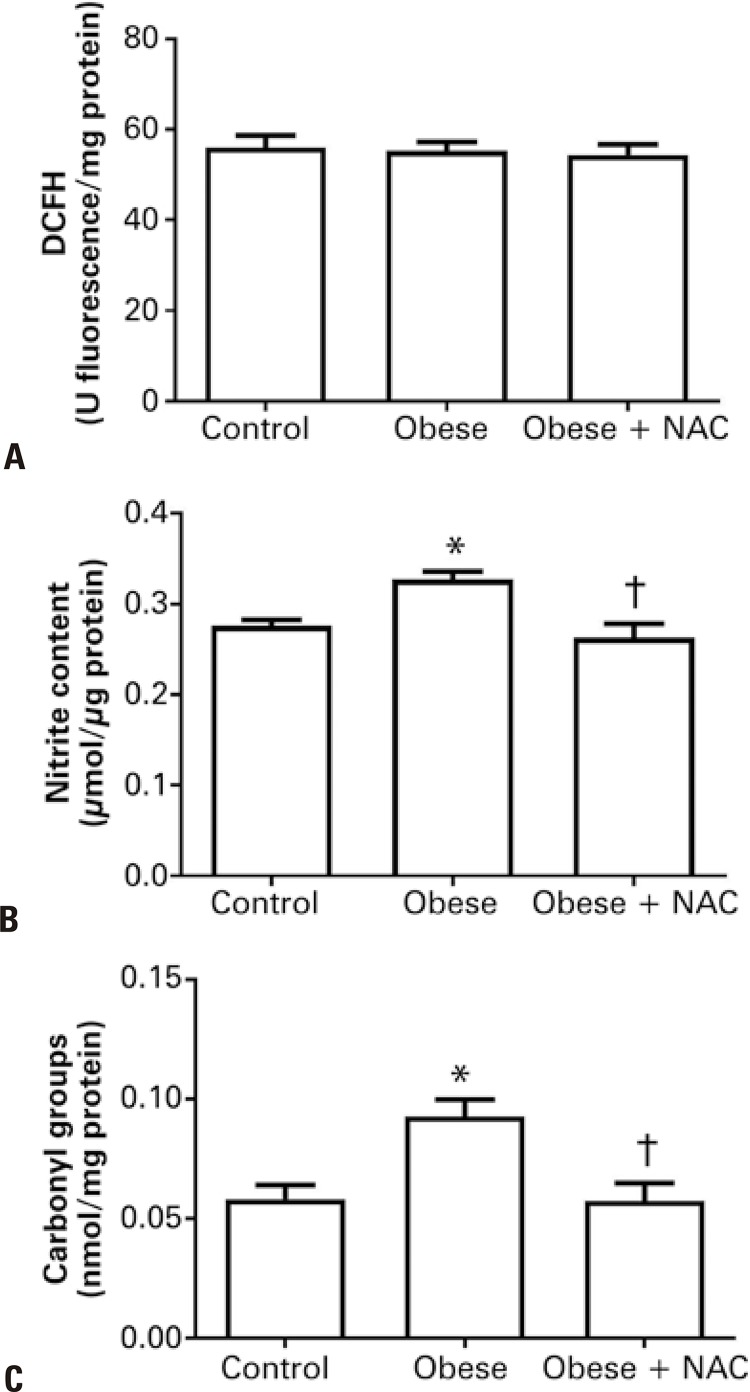
Effects of the administration of N-acetylcysteine on the levels of dichlorofluorescein, nitrite and carbonyl on the cardiac tissue of obese mice * p<0.05 *versus* Control Group; ^†^ p<0.05 *versus* Obese Group. DCFH: dichlorohydrofluorescein; NAC: Obese Group Treated with N-acetylcysteine.

The effects of the antioxidant NAC on the production of reactive species through the techniques DCF and nitric oxide (NO,) and on the oxidative damage over the cardiac tissue of obese mice, are better illustrated in figure 2 . No difference between the three groups was found regarding the levels of DCF ( Figure 2A ). However, there was an increase in nitrite levels in the Obese Group, and a reduction in nitrite levels when the Obese Group received NAC ( Figure 2B ).

### Antioxidant defense and oxidative damage

**Figure 3:**
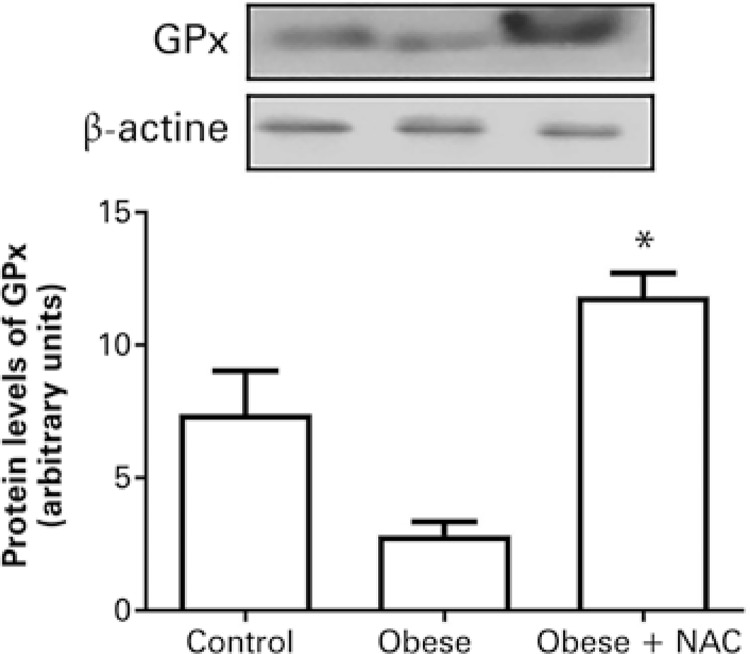
Effects of the administration of N-acetylcysteine on the protein levels of glutathione peroxidase in the cardiac tissue of obese mice * p<0.05 *versus* Obese Group. GPx: glutathione peroxidase; NAC: Obese Group Treated with N-acetylcysteine.

Carbonyl was evaluated as a marker for oxidative damage. There was an increase in protein carbonylation in the Obese Group in comparison to the Control Group. The use of NAC significantly reduced this parameter ( Figure 2C ). Similar results were observed in the analysis of the expression of GPx. The protein levels of GPx were not significantly altered in the Obese Group. However, the administration of the antioxidant NAC significantly elevated the protein levels of this enzyme ( Figure 3 ).

### Effects of N-acetylcysteine on insulin signaling

**Figure 4:**
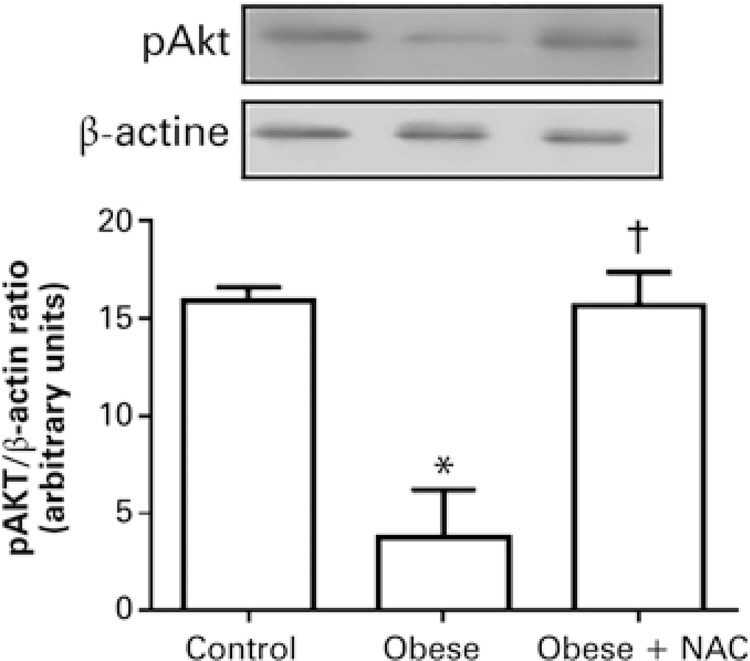
Effects of the administration of N-acetylcysteine on the protein levels of phosphorylated protein kinase B in the cardiac tissue of obese mice * p<0.05 *versus* Control Group; ^†^ p<0.05 *versus* Obese Group. pAKT: phosphorylated protein kinase B; NAC: Obese Group Treated with N-acetylcysteine.

Evaluations were conducted on the protein levels of phosphorylated Akt, as a marker of the insulin transduction pathway. The results showed that obesity significantly reduced the protein levels of Akt phosphorylation in comparison to the Control Group. On the other hand, treatment with NAC was effective in elevating phosphorylated Akt levels in the cardiac tissue to values similar to the Control Group ( Figure 4 ).

## DISCUSSION

This study investigated oxidative stress and its influence on insulin signaling in the myocardium of obese mice. Methodologically, the option chosen to achieve that was to treat the animals with the antioxidant NAC.

Our results show that the treatment with NAC was able to mitigate the production of reactive species, decrease the oxidative damage to the proteins, elevate the protein levels of GPx and alter the phosphorylation on the Akt protein (a crucial protein involved in insulin signaling). These results suggest that, with obesity, there is an increase in oxidative stress, which can affect insulin cell signaling, at least in the myocardium of obese mice.

We used the high-fat diet as a model to induce obesity. ^( 21 )^ In this study, the high fat diet was efficient in elevating the body weight in the Obese Group. The administration of the antioxidant NAC did not significantly alter the body weight of these animals. On the other hand, when NAC was administered concomitantly to the high fat diet, Ma et al., ^( 22 )^ observed a reduction in body weight of the Group Treated with NAC, as compared to the non-treated obese group.

Obese animals presented higher levels of fasting blood glucose in comparison to the Control Group. The increase in fasting blood glucose in obese animals suggested the induction of resistance to insulin. When insulin tolerance was tested, we saw that insulin sensitivity was significantly decreased in the Obese Group. It was interesting that, when supplemented with NAC, there was a reduction in the fasting blood glucose and an increase in insulin sensitivity. Zheng et al. ^( 23 )^ administered NAC orally in the drinking water for 5 months showed similar results. Obese mice that received NAC for 5 months had an improvement in fasting blood glucose and higher glucose tolerance, which reinforces the involvement of oxidative stress in the pathophysiology of insulin resistance.

Zafirovic et al., ^( 24 )^ showed that obesity, in addition to hindering glucose uptake and insulin signaling in the heart, elevates the production of NO in the heart through the increase in the expression of inducible nitric oxide synthase (iNOS) ^( 24 )^ Nitric oxide is an important vasodilator and directly influences cardiac contractility. ^( 25 )^ However, from a biochemical point of view, NO can react with the superoxide anion, preventing it from being dismuted by superoxide dismutase (SOD) in H _2_ O _2_ . In this study, although no significant alterations were observed in the DCFH level, there was an increase in the reactive species of the Obese Group (at least nitrogen), and NAC administration reduced these parameters.

Due to NO ability to react with the superoxide anion, there is a detour in the reaction, which favors the productions of nitrite peroxide through NO, and the formation of H _2_ O _2_ decreases (the main reagent in the dichlorohydrofluorescence technique). Therefore, it is possible that the maintenance of DCFH levels happens exactly because of this detour in the reaction, caused by the increase of NO. In addition, although the activity of direct neutralization of NAC reactive species (scavenger) is significantly lower than the action of antioxidant enzymes, such as SOD, catalase, and GPx, NAC can also have this function and prevent the development of oxidative stress. ^( 15 )^

The increase of reactive species is related to the oxidative damage to proteins, lipids, and nucleic acids. To find out if obesity is related to an increase in protein damage, we evaluated the carbonyl content in the cardiac tissue, which was elevated in the Obese Group. When analyzing the cardiac tissue of obese mice, Li et al., ^( 26 )^ showed that obesity causes an increase of oxidative damage to proteins. ^( 26 )^ According to Guo et al., ^( 27 )^ obesity can significantly elevate the expression of subunits of the complex nicotinamide adenine dinucleotide phosphate oxidase (NADPH oxidase), the main ROS generator in the cardiac tissue. ^( 27 )^ By using NAC, these authors observed a lower translocation of the subunit p67 to the membrane and concluded that NAC administration reduces damage to protein by reducing NADPH oxidase activity in the cardiac tissue of obese rats.

In this study, protein levels were evaluated as indicators of antioxidant activity. Obesity reduced the protein levels of GPx, although not significantly. Treatment with NAC was able to elevate the protein levels of this enzyme. According to Lavoie et al., ^( 28 )^ the oral administration of NAC increases cysteine levels and, consequently, elevates the levels of reduced glutathione (GSH), a tripeptide composed by glycine, glutamyl and cysteine group. ^( 14 , 15 )^ While studying the antioxidant defense system in obese animals, Whiting et al., ^( 29 )^ observed an increase in the production of free radicals in obese hyperglycemic rats, which was related to lower levels of plasma and erythrocyte GPx. ^( 29 )^ In addition, a study conducted by Ballal et al., ^( 30 )^ showed reduced levels of GPx in the cardiac tissue of obese rats. ^( 30 )^ Some studies have shown that an increase in oxidative stress can hinder insulin signaling. ^( 31 - 33 )^ However, there is also a study showing the increase of ROS can elevate insulin sensitivity. ^( 34 )^ These contradictory findings in the literature suggest the relation between oxidative stress and insulin sensitivity is not yet clear and warrants further investigation.

In this study, by using the phosphorylation of Akt as a marker of insulin signaling, it was possible to observe that obesity significantly reduced Akt expression in the cardiac tissue of obese mice. By studying the myocardium of rodents, DeBosch et al., ^( 35 )^ and Shiojima et al., ^( 36 )^ reported that Akt activity can be regulated by insulin, by the nutritional status, and by the redox state. Using Akt phosphorylation as a myocardium marker for insulin resistance, Whaley-Connell et al., ^( 37 )^ reported that the production of oxidants is inversely associated to insulin sensitivity. ^( 37 )^ In order to verify this information, the obese mice were given NAC, which significantly elevated pAKT expression in relation to the Obese Group. Although the exact mechanism of how this occurs is not clear in the literature, it is believed that the increase in the inflammatory process, which is very present in obesity, can increase oxidative stress and hinder insulin signaling in the heart, and this correlation is reinforced in this study through the administration of the antioxidant NAC.

## CONCLUSION

Our results suggested that obesity and/or a high fat diet lead to oxidative stress and resistance to insulin in the cardiac tissue of obese mice. The use of N-acetylcysteine as a methodological and therapeutic strategy suggests a relation between them. However, further studies are necessary to evaluate the cause-effect relation between oxidative stress and resistance to insulin and the possible resulting physiological alterations.
