# Supplementary material for: Reduction of oxidative stress improves insulin signaling in cardiac tissue of obese mice
Source: Einstein (Sao Paulo). 2020 Mar 13;18:eAO5022. doi: 10.31744/einstein_journal/2020AO5022 (PMC7069732; doi:10.31744/einstein_journal/2020AO5022)
Supplement: Translation

# Translation: Redução do estresse oxidativo melhora a sinalização da insulina em tecido cardíaco de camundongos obesos

**Authors:** Matheus Scarpatto Rodrigues, Bruno Luiz da Silva Pieri, Gustavo de Bem Silveira, Rubya Pereira Zaccaron, Ligia Milanez Venturini, Vitor Hugo Comin, Karine Damian Luiz, Paulo Cesar Lock Silveira

**Affiliations:** 1 Brasil Universidade do Extremo Sul Catarinense, Criciúma, SC, Brasil .

**Keywords:** Obesidade, Resistência à insulina, Estresse oxidativo, Miocárdio, Camundongos

## Abstract

**Objetivo:**

Avaliar os efeitos do estresse oxidativo sobre a sinalização da insulina em tecido cardíaco de camundongos obesos.

**Métodos:**

Utilizaram-se 30 camundongos *Swiss* subdivididos igualmente (n=10) em três grupos: Grupo Controle, Grupo Obeso e Grupo Obeso Tratado com N-acetilcisteína. Após estabelecidas a obesidade e a resistência à insulina, os camundongos obesos foram tratados diariamente, durante 15 dias, via gavagem oral, com N-acetilcisteína na dose de 50mg/kg.

**Resultados:**

Observaram-se maiores níveis de glicose sanguínea, conteúdos de nitrito e carbonil, e menores níveis proteicos de glutationa peroxidase e proteína quinase B fosforilada no Grupo Obeso quando comparado a seu respectivo controle. Por outro lado, o tratamento com N-acetilcisteína se mostrou eficiente em diminuir os níveis glicêmicos, os conteúdos de nitrito e carbonil, e aumentar significativamente os níveis proteicos de glutationa peroxidase e proteína quinase B fosforilada, quando comparados ao Grupo Obeso.

**Conclusão:**

Obesidade e/ou dieta hiperlipídica levam a estresse oxidativo e à resistência à insulina no tecido cardíaco de camundongos obesos, e o uso da N-acetilcisteína como estratégia metodológica e terapêutica sugeriu haver relação entre ambos.

## INTRODUÇÃO

A obesidade é um problema de saúde pública mundial e multiétnico, que acomete homens e mulheres de todas as faixas etárias e classes sociais.^( 1 , 2 )^Dados epidemiológicos reforçam a prevalência desta patologia na população mundial. Segundo levantamento da Organização Mundial da Saúde (OMS), realizado em 2016, mais de 1,9 bilhão de adultos sofriam com o sobrepeso, sendo obesos 650 milhões destes.^( 3 )^Com a obesidade, surgem alterações fisiopatológicas importantes, como o *diabetes mellitus* do tipo 2, a resistência à insulina,^( 4 )^além de doenças cardiovasculares e complicações clínicas associadas.^( 5 )^

Várias estratégias têm sido utilizadas para se estudar experimentalmente a obesidade, com destaque para o modelo de indução por dieta rica em gordura (dieta hiperlipídica).^( 6 , 7 )^Camundongos alimentados com tal dieta apresentaram problemas cardíacos significativos, como fibrose do miocárdio, hipertrofia de cardiomiócitos e diminuição da capacidade contrátil do coração.^( 8 )^Camundongos obesos apresentam prejuízos nas captações de glicose e redução da sensibilidade à insulina no miocárdio.^( 9 )^

Vários mecanismos podem estar envolvidos na instalação da resistência à insulina neste órgão, e o elevado estresse oxidativo pode ser um deles. Animais tratados com dieta hiperlipídica apresentam superprodução de espécies reativas do oxigênio (ERO) no fígado e no tecido adiposo.^( 10 )^O miocárdio de camundongos obesos por dieta rica em gordura apresenta aumento de estresse oxidativo.^( 11 )^No entanto, os estudos não são conclusivos e merecem maiores investigações.

Estrategicamente, o uso de um clássico antioxidante poderia melhor demonstrar a relação estresse oxidativo e ação da insulina, e ser de grande valia. Sendo assim, o N-acetilcisteína (NAC), um antioxidante não enzimático derivado do aminoácido cisteína, de fórmula química C_5_H_9_NO_3_S e peso molecular 163,2kDa,^( 12 )^é uma substância que, devido a sua ação antioxidante, pode ser utilizada experimentalmente para melhor demonstrar esta relação.

O NAC é um composto muito empregado na prática clínica como agente mucolítico, para o tratamento de overdoses de paracetamol e prevenção de geração de radicais livres por substâncias tóxicas.^( 13 )^Sua atividade antioxidante está relacionada principalmente à redução do aminoácido extracelular cistina ao aminoácido intracelular cisteína, e à doação de grupamentos tiol para a glutationa reduzida.^( 14 )^Além disso, o NAC pode promover a neutralização direta de ERO como o radical hidroxila e o ácido hipocloroso, prevenindo a instalação do estresse oxidativo e suas possíveis consequências sobre resistência à insulina.^( 15 )^

## OBJETIVO

Analisar o aumento de estresse oxidativo e resistência à insulina em miocárdio de camundongos obesos por dieta rica em gordura. Em caso de resposta positiva, analisar se o uso do N-acetilcisteína mostra alguma relação causal entre tais mecanismos.

## MÉTODOS

### Aspectos étnicos e caracterização dos animais

Foram utilizados, neste estudo, 30 camundongos *Swiss* machos com 45 dias de vida, provenientes do Centro de Bioterismo da UNESC, divididos inicialmente em dois grupos: 10 animais alimentados com dieta padrão para roedores (Grupo Controle) e os demais 20 animais alimentados com dieta hiperlipídica, após comprovada a obesidade e a resistência a insulina, foram subdivididos em dois outros grupos experimentais: Grupo Obeso (n=10) e Grupo Obeso Tratado com N-acetilcisteína (n=10). Todos os animais foram mantidos em ciclos de 12 horas claro/escuro, ambiente com 70% de umidade e temperatura entre 20°C e 22°C, alojados em gaiolas de poliuretano com cobertura metálica (um animal por caixa), e alimentados por 12 semanas com ração padrão (carboidrato: 70%; proteína: 20%; gordura: 10%; totalizando 3,8kcal/g) ou ração hiperlipídica (carboidrato: 38,5%; proteína: 15%; gordura: 46,5%; totalizando 5,4kcal/g) para roedores e água *ad libitum* .

Este estudo foi avaliado e aprovado pelo Comitê de Ética no Uso de Animais (CEUA) da Universidade do Extremo Sul Catarinense (UNESC), protocolo 042/2016-2. Todos os experimentos respeitaram estritamente os princípios éticos da experimentação animal.

### Tratamento dos animais com antioxidante N-acetilcisteína

Após a instalação da obesidade e da resistência à insulina, os animais que receberam previamente a dieta hiperlipídica foram subdivididos em dois grupos: Grupo OB, grupo de obesos alimentados com dieta hiperlipídica (n=10), e Grupo OB + NAC, grupo de obeso tratado durante 15 dias com N-acetilcisteína (n=10). Vale ressaltar que os animais deste último grupo só receberam a terapia antioxidante após a devida comprovação da obesidade e da resistência à insulina, cujas avaliações foram devidamente realizadas após três meses de exposição dos animais à dieta rica em gordura. A NAC foi administrada uma vez ao dia, via gavagem oral (50mg/kg), durante 15 dias. O estudo foi realizado nos meses de fevereiro e março de 2017. Ao todo, a duração do protocolo experimental (período experimental) foi de 14 semanas e 1 dia. Depois de 24 horas da última administração da NAC, os animais foram eutanasiados, e o coração foi extraído, para posterior realização das técnicas bioquímicas.

### Glicemia em jejum

O teste foi realizado ao final do período experimental. A alimentação foi retirada 6 horas antes do teste. A dosagem da glicose sanguínea foi feita por meio de glicosímetro, e os resultados foram expressos em mg/dL.

### Teste de tolerância à insulina

O teste de tolerância à insulina foi realizado antes do início da administração de antioxidante e ao final do período experimental. O alimento foi retirado 6 horas antes do teste, e a primeira coleta sanguínea equivale ao tempo zero. Após isso, a insulina (2U/kg de peso corporal) foi injetada intraperitonealmente, e amostras de sangue foram coletadas pela cauda nos tempos 5, 10, 15, 20, 25 e 30 minutos, sendo a determinação da glicemia realizada com um glicosímetro. A velocidade constante do decaimento da glicose (K_RI_) foi calculada usando a fórmula 0,693/t_1/2_. O t_1/2_da glicose foi calculado a partir da curva da análise dos mínimos quadrados da concentração da glicose sérica, durante a fase de decaimento linear. Esse teste foi realizado para comprovação da instalação da resistência à insulina nos camundongos obesos e verificar possível melhora nos animais tratados.

### Western blotting

Após 24 horas da última administração de NAC, os animais sofreram eutanásia, e o ápice cardíaco foi extraído e imediatamente homogeneizado em tampão específico, contendo inibidores de proteases e fosfatases. A concentração de proteínas totais fora determinada segundo método de Bradford et al.^( 16 )^As proteínas foram ressuspensas e conservadas em tampão Laemmli, contendo 100mmol/L de ditiotreitol (DTT),^( 17 )^e realizou-se a determinação do *immunoblotting* com anticorpos específicos. Para isso, alíquotas contendo 250µg de proteína por amostra foram aplicadas sobre gel de poliacrilamida (SDS-PAGE) e, posteriormente, foram transferidas para membranas de nitrocelulose via eletroforese. A seguir, as membranas foram incubadas com anticorpos primários específicos anti-pAKT, glutationa peroxidase (GPx) e beta-actina adquiridos da Santa Cruz Biotechnology (Santa Cruz, California, USA), sob agitação constante, durante a noite, a 4°C. Posteriormente, as membranas foram incubadas em solução com anticorpo secundário conjugado com peroxidase, durante 2 horas, à temperatura ambiente. Após, as membranas foram incubadas por 2 minutos em substrato enzimático e expostas ao filme de raios X em cassete de revelação radiográfica. A intensidade das bandas foi determinada por meio da leitura das radiografias reveladas por densitometria ótica, utilizando um scanner (HP G2710) e o programa ImageJ.

### Diclorohidrofluoresceína

A oxidação do 2’7’diacetato de diclorohidrofluoresceína (DCFH-DA) pelas células causa a fluorescência da difluoresceína (DCF), que pode facilmente ser lida em espectrofotômetro. Neste ensaio, 100µL de água e 75µL de DCFH-DA foram adicionados a 25µL de homogeneizado de amostra, homogeneizados em vórtex e levados ao banho-maria a 37°C ao abrigo da luz, por um período de 30 minutos. Separadamente, foi preparada a curva de calibração, onde se utilizou como padrão o DCFH-DA 0,1µM diluído em tampão fosfato/EDTA em pH 7,4 em diferentes concentrações. Tanto as amostras quanto a curva de calibração foram processadas em duplicata e ao abrigo da luz. Ao final dos 30 minutos, foram feitas as leituras no espectrofotômetro (525nm excitação e 488nm de emissão), sendo os resultados expressos em nmol de DCF por mg de proteínas.^( 18 )^

### Indicador de formação de óxido nítrico

Para mensurar o conteúdo de nitrito, as amostras foram incubadas com reagente Griess (1% sulfanilamida e 0,1% de N-1 (naftil) etilenodiamina) em temperatura ambiente, por 10 minutos, e a absorbância foi medida a 540nm. O conteúdo de nitritos foi calculado com base em uma curva padrão de 0 a 100nM realizada com o metabólito nitrito de sódio (NaNO2). Os resultados foram calculados em µmol nitrito/mg proteína.^( 19 )^

### Carbonilação de proteínas

O conteúdo de carbonilas foi determinado espectrofotometricamente a 370nm como previamente descrito por Levine et al.^( 20 )^Os resultados foram calculados como nmol/mg de proteína empregando o coeficiente de extinção molar de dinitrofenilhidrazonas de 22.000M^-1^cm^-1^.

### Análises estatísticas

Os resultados foram expressos como média±erro padrão da média e analisados estatisticamente por meio de análise de variância (ANOVA), seguida do teste *post hoc* Bonferroni. Foi adotado nível de significância p<0,05. A análise estatística foi realizada por meio do software GraphPad Prism^®^(versão 5.00) para Microsoft Windows^®^.

## RESULTADOS

### Peso corporal e glicemia em jejum

A avaliação dos efeitos da suplementação de NAC sobre o peso corporal, da glicemia em jejum e da tolerância à insulina em camundongos obesos está ilustrada na figura 1 . Como esperado, a alimentação dos camundongos com dieta hiperlipídica foi eficiente em aumentar o peso corporal dos camundongos; a administração do antioxidante NAC não alterou tal parâmetro nos camundongos, o qual permaneceu elevado de modo significativo em relação ao Grupo Controle ( Figura 1A). Os níveis glicêmicos em jejum dos camundongos obesos foram maiores quando comparados ao Controle. A administração de NAC reduziu os níveis glicêmicos de jejum no Grupo Tratado com NAC, em relação ao Grupo Obeso ( Figura 1B).

**Figura 1:** Efeitos da suplementação de N-acetilcisteína sobre o peso corporal, glicemia em jejum e tolerância à insulina em camundongos obesos * p<0,05 *versus* Grupo Controle;^†^p<0,05 *versus* Grupo Obeso. NAC: Grupo Obeso Tratado com N-acetilcisteína; KRI: velocidade constante do decaimento da glicose.

### Teste de tolerância à insulina

Para avaliar-se a sensibilidade à insulina entre os grupos estudados, utilizou-se a constante de decaimento da glicemia (k_RI_). A obesidade reduziu significativamente os valores de k_RI_quando comparado ao Grupo Controle. Por outro lado, o tratamento com antioxidante NAC reverteu esse efeito significativamente ( Figura 1C).

### Produção de espécies reativas no tecido cardíaco

Os efeitos do antioxidante NAC sobre a produção de espécies reativas pelas técnicas de DCF e óxido nítrico (NO), e sob o dano oxidativo no tecido cardíaco de camundongos obesos, estão mais bem ilustrados na figura 2 . Nenhuma diferença entre os três grupos foi encontrada quanto aos níveis de DCF (Figura 2A). No entanto, observaram-se aumento da concentração de nitrito no Grupo Obeso e redução desta concentração quando o Grupo Obeso recebeu a NAC (Figura 2B).

**Figura 2:** Efeitos da administração de N-acetilcisteína sobre os níveis de diclorofluoresceína, nitrito e carbonil no tecido cardíaco de camundongos obesos * p<0,05 *versus* Grupo Controle;^†^p<0,05 *versus* Grupo Obeso. DCFH: Diclorohidrofluoresceína; NAC: Grupo Obeso Tratado com N-acetilcisteína.

### Defesa antioxidante e dano oxidativo

Avaliou-se carbonil como marcador de dano oxidativo. Observou-se aumento na carbonilação de proteínas no Grupo Obeso quando comparado ao Controle. O uso de NAC reduziu significativamente este parâmetro (Figura 2C). Resultados similares foram observados quando se analisou a expressão da GPx. Os níveis proteicos de GPx não se alteraram significativamente no Grupo Obeso. No entanto, a administração do antioxidante NAC elevou marcantemente os níveis proteicos dessa enzima ( Figura 3 ).

**Figura 3:** Efeitos da administração de N-acetilcisteína sobre os níveis proteicos de glutationa peroxidase no tecido cardíaco de camundongos obesos * p<0,05 *versus* Grupo Obeso. GPx: glutationa peroxidase; NAC: Grupo Obeso Tratado com N-acetilcisteína.

### Efeitos do N-acetilcisteína sob a sinalização da insulina

Avaliaram-se os níveis proteicos de Akt fosforilada, como marcador da via de transdução da insulina. Os resultados mostraram que a obesidade reduziu significativamente os níveis proteicos da fosforilação da Akt, quando comparado ao Grupo Controle. Por outro lado, o tratamento com NAC mostrou-se eficaz em elevar os níveis da Akt fosforilada no tecido cardíaco a valores similares ao Grupo Controle ( Figura 4 ).

**Figura 4:** Efeitos da administração de N-acetilcisteína sobre os níveis proteicos da proteína quinase B fosforilada no tecido cardíaco de camundongos obesos *p<0,05 *versus* Grupo Controle; † p<0,05 *versus* Grupo Obeso. pAKT: proteína quinase B fosforilada; NAC: Grupo Obeso Tratado com N-acetilcisteína.

## DISCUSSÃO

O presente estudo investigou o estresse oxidativo e sua influência sobre a sinalização da insulina em miocárdio de camundongos obesos. Metodologicamente, optou-se, para isso, tratar os animais com o antioxidante NAC.

Nossos resultados mostram que o tratamento com NAC foi capaz de atenuar a produção de espécies reativas, diminuir danos oxidativos às proteínas, elevar os níveis proteicos de GPx e ainda alterar a fosforilação da proteína Akt (proteína crucial envolvida na sinalização da insulina). Tais resultados permitem sugerir que, na obesidade, ocorre aumento do estresse oxidativo, o que pode influenciar e afetar a sinalização celular insulínica, ao menos no miocárdio de camundongos obesos.

Utilizamos a dieta hiperlipídica como modelo para indução da obesidade.^( 21 )^No presente estudo, a dieta rica em gordura foi eficiente em elevar o peso corporal nos animais do Grupo Obeso. A administração do antioxidante NAC não alterou significativamente o peso corporal desses animais. Por outro lado, quando NAC foi administrada de forma concomitante à oferta da dieta rica em gordura, Ma et al.,^( 22 )^observaram redução do peso corporal do Grupo Tratado com NAC, comparado com o Grupo Obeso não tratado.

Animais obesos apresentaram maiores níveis sanguíneos de glicose em jejum quando comparado ao Grupo Controle. O aumento da glicemia em jejum nos animais obesos sugeriu instalação da resistência corporal à insulina. Ao realizar o teste de tolerância à insulina, verificou-se que a sensibilidade à insulina foi, de fato, significativamente diminuída no Grupo Obeso. Interessantemente, quando suplementado com NAC, houve redução da glicemia em jejum, bem como aumento da sensibilidade à insulina. Estudo desenvolvido por Zheng et al.,^( 23 )^administrando o NAC por 5 meses via oral diluído na água de beber mostrou resultados similares. Camundongos obesos que receberam NAC por 5 meses tiveram melhora no quadro glicêmico de jejum, bem como melhor tolerância à glicose, evidência que reforça a participação do estresse oxidativo sob a fisiopatologia da resistência à insulina.

Zafirovic et al.,^( 24 )^mostraram que a obesidade, além de prejudicar a captação de glicose e a sinalização da insulina no coração, eleva, via aumento da expressão de oxido nítrico sintase induzível (iNOS), a produção de NO neste órgão.^( 24 )^O NO é um importante vasodilatador e influencia diretamente na contratilidade cardíaca.^( 25 )^Porém, no ponto de vista bioquímico, o NO pode reagir com o ânion superóxido o impedindo de ser dismutado pela superóxido dismutase (SOD) em H_2_O_2_. No presente estudo, embora não tenham sido observadas alterações significativas no nível de DCFH, houve aumento de espécies reativas no Grupo Obeso (ao menos de nitrogênio), e a administração de NAC reduziu tais parâmetros.

Devido à capacidade de reação do NO com o ânion superóxido, ocorre desvio na reação, que favorece a produção de peróxido nitrito via NO e diminui a formação de H_2_O_2_(principal reagente na técnica do diclorohidrofluoresceína). Assim, é possível que a manutenção dos níveis de DCFH seja justamente devida ao fato de haver este desvio na reação provocado pelo aumento de NO. Em adição, embora a atividade de neutralização direta de espécies reativas ( *scavenger)* do NAC seja significativamente menor do que a ação das enzimas antioxidantes como SOD, catalase e GPx, o NAC também pode exercer esta função e prevenir o desenvolvimento de estresse oxidativo.^( 15 )^

O aumento de espécies reativas relaciona-se com o dano oxidativo a proteínas, lipídeos e ácidos nucleicos. Para saber se a obesidade está relacionada ao aumento de dano proteico, avaliou-se o conteúdo de carbonil no tecido cardíaco, o qual estava elevado no Grupo Obeso. De fato, analisando o tecido cardíaco de camundongos obesos, Li et al.,^( 26 )^demonstraram que, na obesidade, ocorre aumento de dano oxidativo em proteínas.^( 26 )^Segundo Guo et al.,^( 27 )^a obesidade é capaz de elevar significativamente a expressão de subunidades do complexo fosfato de dinucleótido de nicotinamida e adenina oxidase (NADPH oxidase), principal gerador de ERO no tecido cardíaco.^( 27 )^Utilizando o NAC, estes autores observaram menor translocação da subunidade p67 para a membrana e concluíram que a administração de NAC reduz danos à proteína por meio da redução da atividade da NADPH oxidase em tecido cardíaco de ratos obesos.

No presente estudo, avaliaram-se os níveis proteicos da GPx como indicativo de atividade antioxidante. A obesidade reduziu os níveis proteicos de GPx, embora não de maneira significante. O tratamento com NAC foi capaz de elevar os níveis proteicos desta enzima. Segundo Lavoie et al.,^( 28 )^a administração oral de NAC aumenta os níveis de cisteína e, consequentemente, eleva os níveis de glutationa reduzida, a GSH, um tripeptídeo composto por glicina, glutamil e grupamento cisteína.^( 14 , 15 )^Estudando o sistema de defesa antioxidante em animais obesos, Whiting et al.,^( 29 )^observaram aumento na produção de radicais livres em ratos obesos hiperglicêmicos, o que foi relacionado com menores níveis de GPx plasmática e eritrocitária.^( 29 )^Em adição, estudo realizado por Ballal et al.,^( 30 )^mostrou reduzidos níveis de GPx em tecido cardíaco de ratos obesos.^( 30 )^Alguns estudos têm demonstrado que o aumento do estresse oxidativo pode prejudicar a sinalização da insulina.^( 31 - 33 )^No entanto, há, também, estudo demonstrando que o aumento de ERO pode elevar a sensibilidade à insulina.^( 34 )^Esses achados contraditórios na literatura sugerem que a relação entre estresse oxidativo e sensibilidade à insulina ainda não está elucidada e merece maiores investigações.

No presente estudo, utilizando a fosforilação da Akt como marcador da sinalização da insulina, observou-se que a obesidade reduziu significativamente a expressão da Akt no tecido cardíaco de camundongos obesos. Estudando a Akt no miocárdio de roedores, DeBosch et al.,^( 35 )^e Shiojima et al.,^( 36 )^relataram que a atividade desta proteína pode ser regulada pela insulina, pelo estado nutricional e pelo estado redox. Usando a fosforilação de Akt como marcador miocárdico de RI, Whaley-Connell et al.,^( 37 )^reportaram que a produção de oxidantes está inversamente associada à sensibilidade à insulina.^( 37 )^Com o intuito de verificar esta informação, administrou-se, nos camundongos obesos, o antioxidante NAC, elevando-se, assim, a expressão da proteína pAKT de modo significativo em relação ao Grupo Obeso. Embora o exato mecanismo de como isso ocorra não esteja claro na literatura, acredita-se que o aumento do processo inflamatório, muito presente na obesidade, possa elevar o estresse oxidativo e prejudicar a sinalização da insulina neste órgão − correlação esta reforçada neste estudo por meio da administração do antioxidante NAC.

## CONCLUSÃO

Nossos resultados sugerem que a obesidade e/ou a dieta hiperlipídica levam ao estresse oxidativo e à resistência à ação da insulina no tecido cardíaco de camundongos obesos. O uso de N-acetilcisteína como estratégia metodológica e terapêutica sugere relação entre ambos. No entanto, futuros estudos são necessários para avaliar-se a relação causa-efeito entre estresse oxidativo e resistência à insulina, bem como as possíveis alterações fisiológicas resultantes.
